# Welding of silver nanowire networks via flash white light and UV-C irradiation for highly conductive and reliable transparent electrodes

**DOI:** 10.1038/srep32086

**Published:** 2016-08-24

**Authors:** Wan-Ho Chung, Sang-Ho Kim, Hak-Sung Kim

**Affiliations:** 1 Department of Mechanical Convergence Engineering Hanyang University, 17 Haendang-Dong, Seongdong-Gu, Seoul 133-791 South Korea; 2 Nanotech and beyond Co., 125-10, Techno 2-ro, Yuseong-gu, Daejeon, 34024, South Korea; 3 Institute of Nano Science and Technology, Hanyang University, Seoul, 133-791, South Korea

## Abstract

In this work, silver nanowire inks with hydroxypropyl methylcellulose (HPMC) binders were coated on polyethylene terephthalate (PET) substrates and welded via flash white light and ultraviolet C (UV-C) irradiation to produce highly conductive transparent electrodes. The coated silver nanowire films were firmly welded and embedded into PET substrate successfully at room temperature and under ambient conditions using an in-house flash white light welding system and UV-C irradiation. The effects of light irradiation conditions (light energy, irradiation time, pulse duration, and pulse number) on the silver nanowire networks were studied and optimized. Bending fatigue tests were also conducted to characterize the reliability of the welded transparent conductive silver nanowire films. The surfaces of the welded silver nanowire films were analyzed via scanning electron microscopy (SEM), while the transmittance of the structures was measured using a spectrophotometer. From the results, a highly conductive and transparent silver nanowire film with excellent reliability could be achieved at room temperature under ambient conditions via the combined flash white light and UV-C irradiation welding process.

Transparent conductive electrodes (TCEs) with high electrical conductivity and good optical transparency have been widely used in numerous flexible electronic devices, such as touch screens^1^,^2^, active-matrix LCDs^3^,^4^, light emitting diodes^5^,^6^, and solar cells^7^. Various materials have been employed to prepare TCEs, including indium tin oxide (ITO)^8^,^9^, fluorine-doped tin oxide (FTO)^10^,^11^, graphene^12^,^13^, carbon nanotubes (CNTs)^1^,^14^, and metal nanowires (e.g. silver and copper)^15^,^16^,^17^. Among these, ITO has found the most widespread use in TCEs. However, the material has several drawbacks due to its brittleness, high material cost, and the scarcity of indium^16^,^18^. The brittleness of ITO makes it a particularly unsuitable choice for flexible electronic applications. In contrast, graphene and CNTs have attracted significant attention for their bending characteristics. Unfortunately, these materials also exhibit a higher sheet resistance (100~1000 Ω/sq) and lower transmittance (<80%) when compared to ITO^19^. To overcome these drawbacks, silver and copper nanowires have been recently utilized to fabricate flexible TCEs due to the low cost, high ductility, and low sheet resistance of the structures^20^,^21^. Also, the fabricated TCEs using silver and copper nanowires were adjusted in the various applications such as light emitting diode, touch screen panel and sola cell with low cost. Therefore, the fabrication of TCEs using silver and copper nanowires could be effected for improvement of the flexible electronics industrial^17^.

The dispersion of the silver nanowire solution is an important factor when coating silver nanowires on a polymer substrate. In general, silver nanowires are easily dispersed in an ethylene glycol, isopropyl alcohol, and deionized (DI) water solution with the addition of organic binders as dispersants and adhesive agents^22^,^23^. The adhesive agent in a silver nanowire solution plays an important role in the sheet resistance uniformity of TCE films^22^,^23^. However, it is difficult to weld silver nanowires with adhesive agents because it can inhibit contact between the individual nanowires. In previous work, silver nanowires have been welded without an adhesive agent, as evaporation of the adhesive agent and welding of the silver nanowires could not be achieved simultaneously by a thermal welding process^24^.

In this work, to resolve these problems, a combined light irradiation strategy was newly developed to weld the silver nanowires with an adhesive agent. In this procedure, both flash white light emitted by a xenon lamp^25^,^26^,^27^,^28^ and UV-C irradiation via a UV-C lamp were employed for welding. Using the combined light irradiation process, which covers a wide wavelength range from the UV-C (180 nm) to infrared (1000 nm) regimes, silver nanowires were successfully welded in a few seconds at room temperature under ambient conditions (Fig. 1). The combined light welding process was composed of two steps; pre-treatment process and main welding process. The role of pre-treatment process was the evaporation of organic binder around silver nanowire and the role of the main welding process was the welding of the silver nanowires. Therefore, to improve the welding characteristic as well as the uniformity of silver nanowire, the combined light welding process was optimized by varying several flash white light and UV-C irradiation conditions, including the irradiation energy, pulse duration, irradiation time, and pulse number. The welded silver nanowire films were subsequently characterized via scanning electron microscopy (SEM) and Fourier transform infrared spectroscopy (FT-IR). The sheet resistance of the silver nanowire films was also measured by a four-probe method, while the transparency of the structures was evaluated with a spectrophotometer.

## Results

The combined flash white light welding process with UV-C irradiation for the silver nanowires TCE films are schematically illustrated in Fig. 2(a). Silver nanowire solutions with hydroxypropyl methylcellulose (HPMC) binder as the adhesive binder (from 0 to 0.3 wt%) were coated on PET substrates using bar-coater method, as described in the experimental section. To analyze the sheet resistance uniformity, the sheet resistance of silver nanowire films was measured into the nine-parts, individually. As shown in the Fig. 2(b), the sheet resistance of the coated silver nanowires decreased as the weight fraction of HPMC binder was increased. Such a result indicates that more extensive contact was induced among the silver nanowires at higher HPMC binder concentrations. Furthermore, the sheet resistance uniformity improved as the weight fraction of HPMC binder was increased with the exception of the film made with 0.3 wt% HPMC binder (Fig. 2(b,c), Table 1). These findings are similar to those reported by other researchers, where methoxyl and hydroxypropyl groups in the HPMC binder were found to improve dispersion and adhesion among the silver nanowires^23^,^29^,^30^. In the case of the silver nanowire film prepared with 0.3 wt% HPMC, the standard deviation of the sheet resistance became higher than that of the other samples. This result may be attributed to silver nanowire agglomeration due to the use of an exceedingly high ratio of HPMC binder (Fig. 2(c,d)). Therefore, the weight fraction of HPMC binder was maintained at 0.15% for the remainder of this work.

The coated silver nanowire films were dried using an NIR lamp followed by UV-C light irradiation. The UV-C irradiation time was varied from 60 sec to 300 sec with a power of 2.78 mW/cm^2^. After the UV-C light irradiation, the main flash white light welding process (energy: 26 J/cm^2^, on-time:10 ms and pulse number: 1) was conducted. The sheet resistance of the silver nanowire films decreased at longer UV-C irradiation times (Fig. 3(a)). SEM images of the silver nanowires were acquired after the main flash white light welding process with respect to UV-C irradiation durations; the results are shown in Fig. 3(b–e). After UV-C irradiation for 180 sec and 300 sec, the HPMC binder among the silver nanowires completely evaporated and the nanowires were found to be embedded in the PET substrate. Meanwhile, in the case of a 60 sec UV-C irradiation treatment, the silver nanowires were not embedded in the PET substrate and some HPMC binder was still present (Fig. 3(c)). The residual HPMC binder may inhibit direct contact between the silver nanowires and thus, the sheet resistance of the sample irradiated for 60 sec was higher than that of the specimens irradiated for 180 sec and 300 sec before the main flash white light welding step (see Fig. 3(a)). After the main flash white light welding process, the sheet resistance decreased for the silver nanowire film irradiated with UV-C light for 60 sec. In contrast, for the samples subjected to 180 sec and 300 sec of UV-C irradiation, the sheet resistance increased after the main flash white light irradiation step (Fig. 3(a)). Such a result may arise because the 180 sec and 300 sec UV-C irradiation treatments fully embedded the silver nanowires in the PET substrate. Consequently, the main flash white light irradiation step could not weld and connect the silver nanowires. Meanwhile, in the case of a 60 sec UV-C irradiation time, the silver nanowires were not embedded in the PET substrate and thus, the main flash white light irradiation treatment served to weld the silver nanowires and decrease the sheet resistance.

As shown in the FT-IR spectra of no treated Ag nanowire film, there were various peaks that meaning of ring C–H + C = O out of plane bending at 725 cm^−1^, C-O bond at 1180 cm^−1^ and C = O bond at 1730 cm^−1 ^31,32 (Fig. 3(f)). After UV-C irradiation to the Ag nanowire films for 60 sec, the various peaks related to the chemical structures of PET substrate were still existed. However, the most of those peaks were changed with UV-C irradiation for 180 sec. Especially, the peaks of C-O bond and C = O bond in the 1180 cm^−1^ and 1730 cm^−1^ were drastically decreased which means the decomposition of PET substrate^30^,^32^. Generally, it is well known that the carbon and oxygen bond of PET polymer was decomposed by UV-C irradiation with high intensity and long time^33^. Also, the morphology of PET substrate surface was changed with the decomposition of the PET polymer^34^. However, As shown in the AFM image of Fig. 3(g,h), when the UV-C light was irradiated directly on the PET substrate covered with only HPMC except for silver nanowire, the root mean square (Rq) value (1.649 nm) was reduced compared to the Rq value before UV-C light irradiation (3.04 nm). Thus, the topography of silver nanowire films was slightly improved without the damage of PET substrate because the HPMC binder was decomposed by priority before generating the decomposition of the PET substrate. Therefore, the UV-C light was used for the effectively welding of silver nanowire as well as the decomposition of HPMC binder that help the silver nanowire embedded into the PET substrate.

With the UV-C irradiation duration fixed at 60 sec, the UV-C irradiation energy was varied during the pre-treatment step; the resulting sheet resistance of the welded silver nanowire films is displayed in Fig. 4(a). The sheet resistance of the films after the UV-C pre-treatment and main flash white light welding process decreased as the irradiation energy of the UV-C light was increased. This was because the photo-catalytic effect of UV-C light irradiation could decompose the HPMC binder, thereby inducing closer contact among the silver nanowires (Fig. 4(c)). Note that UV light has been used for the decomposition of high molecular weight HPMC compounds^35^,^36^,^37^. However, upon irradiation with UV-C light having an excessively high energy (more than 2.78 mW/cm^2^), the PET substrate was damaged (Fig. 4(b)). The degradation or aging of PET polymer substrate could be occurred by UV-C light irradiation. However, to generate the degradation or aging of PET substrate, UV-C light should be irradiated with strong intensity light or for long time^34^,^38^. After the main flash white light irradiation (energy: 26 J/cm^2^, on-time: 10 ms and pulse number: 1), the sheet resistance decreased significantly as the silver nanowires were firmly welded, as shown in Fig. 4(d). The welding of the silver nanowires may be due to photo-thermal effects generated by the irradiated flash white light during the main flash light welding process. Such photo-thermal effects may be ascribed to surface plasmon resonance, whereby absorbed photons are converted into phonons in the silver nanowires after light exposure^16^,^39^,^40^,^41^. To measure the absorption wavelength of the silver nanowires, a spectrophotometer was employed. As shown in Fig. 4(e), light absorption in the silver nanowires was observed at a wavelength of approximately 355.2 nm. Note that the absorbed wavelength of 355.2 nm lies within the spectrum of flash white light wavelengths, thus the surface plasmon resonance followed by the welding of the silver nanowires junctions could be achieved efficiently (Fig. 4(e,f)).

To enhance further the welding of the silver nanowire films, multi-pulsed flash white light and UV-C irradiation were used simultaneously in the pre-treatment process, as shown in Figs 5(a) and 6(a). Note that in our previous study, the use of multi-pulsed flash white light with a small energy in the pre-treatment process induced gradual evaporation of polymer binder and a smoother surface morphology for the sintered metal films without damaging the polymer substrate^27^. To find an optimal combination of UV-C and the flash white light, the pre-treatment and main welding steps were carried out for two different cases (Figs 5(a) and 6(a)): one is to use only flash light (Fig. 5(a)) and another is to use the flash light and UV-C light simultaneously (Fig. 6(a)).

Figure 5(b) is the sheet resistance of the welded silver nanowire films using only the flash white light irradiation with the pre-treatment (energy: 50 J/cm^2^ to 70 J/cm^2^, pulse duration: 10 ms, irradiation time: 5 ms, pulse number: 15) and main welding (energy: 26 J/cm^2^, irradiation time: 10 ms, pulse number: 1) procedures (case 1 in Fig. 5(a)). The sheet resistance of the silver nanowire films increased at a pre-treatment energy of 50 J/cm^2^, as the silver nanowires became detached due to evaporation of the HPMC binder. Furthermore, as shown in the SEM image of the Fig. 5(c), HPMC binder remained in the silver nanowire films after the flash white light irradiation due to the use of light with insufficient energy. However, after irradiation with 26 J/cm^2^ of flash white light energy in the main welding process, the sheet resistance of the silver nanowire films decreased. This result could be attributed to evaporation of the remaining HPMC binder and welding of the junctions among the silver nanowires (Fig. 5(f)). Upon irradiation with 60 J/cm^2^ of multi-pulse flash white light energy, the sheet resistance of the silver nanowire films also decreased, as sufficient energy was provided to evaporate the HPMC binder (Fig. 5(d)). Consequently, the silver nanowires were easily welded in the main welding process, leading to a decrease in the sheet resistance (Fig. 5(g)). For the case of irradiation with 70 J/cm^2^ of light energy after the pre-treatment process, the sheet resistance of the silver nanowire films decreased significantly (Fig. 5(b)). However, after the main flash white light irradiation step, the sheet resistance of the films increased again. Such a finding may be ascribed to the silver nanowires being slightly welded and embedded in the PET substrate by the pre-treatment process, as shown in the Fig. 5(e). Therefore, after the main welding process, the silver nanowires became twisted and further embedded into the PET film, leading to insufficient welding (Fig. 5(h)). This in turn caused the sheet resistance of the silver nanowire film to increase again. It may thus be concluded that excessive light energy during the pre-treatment could serve to embed the silver nanowires into the PET film, thereby inhibiting nanowire welding in the main flash white light welding process.

In case 2 of Fig. 6(a), UV-C light was continuously applied with the flash white light in both the pre-treatment and main welding processes.

Figure 6(b) shows the sheet resistance of the silver nanowire films welded by flash white light under continuous UV-C irradiation. It was found that the simultaneous UV-C irradiation with the flash white light could weld the silver nanowires and decrease the sheet resistance more efficiently compared to the only flash light cases. It is noteworthy that the welded silver nanowires could be completely embedded into the PET substrate expecting the excellent mechanical reliability (Fig. 6(c–e)). As shown in Fig. 6(b), it was also found that the 50 J/cm^2^ pre-treatment of the flash light with continuous UV-C irradiation could weld the silver nanowires most efficiently compared to other pre-treatment flash light energy cases. It is because higher pre-treatment energy than 50 J/cm^2^ might embed the silver nanowires into PET substrate in the pre-treatment process, which hinder the welding of them in the main sintering process.

To evaluate the reliability of the silver nanowire films welded by flash white light under continuous UV-C irradiation, bending tests were conducted. The outer bending radius was 7 mm during testing, and each sample was subjected to 1000 cycles bending loading at a frequency of 2 Hz. Fig. 7(a) shows the bending test results before and after the combined light welding process. After 300 cycles bending loadings, the ratios of the sheet resistance after bending to that measured before testing were similar for both sets of samples. However, after 300 bending cycles, the sheet resistance ratio of the silver nanowire films without combined light welding increased significantly. It is believed that, before the combined light welding process, the silver nanowires were not embedded in the PET and thus, became detached from the substrate during the severe mechanical bending tests. Meanwhile, only a slight 1.5 times increase was observed in the sheet resistance ratio of the silver nanowire films welded by flash white light under continuous UV-C irradiation.

As shown in the Fig. 7(c), the silver nanowires with HPMC binder were coated on the PET substrate. After combined flash light irradiation, most of silver nanowire was embedded in the PET substrate except the top of partial silver nanowire (Fig. 7(d)). It was shown that the irradiated flash light with UV-C light made the polymer binder decomposed. Therefore, because of the generated high heat in the silver nanowire by photo-thermal and photo-catalytic effect, silver nanowire could be embedded into the PET substrate. Also, to analyze the surface morphology, Rq value was measured. The Rq value after combined flash light irradiation (32.443 nm) was reduced compared to the Rq value before combined flash light irradiation (59.736 nm). This phenomena comes from the decomposition of HPMC organic binder and the embedding of silver nanowire into PET substrate.

The transmittance is also an important factor to consider when evaluating transparent electrodes. Here, the silver nanowire films welded under optimal combined welding conditions exhibited a higher transmittance (average transmittance: 98.76%, reference: PET substrate) when compared (average transmittance: 98.45%, reference: PET substrate) to dried silver nanowire films (Fig. 7(b)). This result may be attributed to effective evaporation of the HPMC binder, whose presence serves to lower the transparency of silver nanowire films. As a result, highly conductive and flexible silver nanowire films with high transparency were successfully prepared on PET substrates (Fig. 7(e)).

## Discussion

From the obtained results, it was concluded that a highly conductive and transparent silver nanowire film with excellent mechanical reliability could be produced at room temperature under ambient conditions via the combined flash white light and UV-C irradiation welding process. The welding of the silver nanowires and their full embedding into PET film could be achieved simultaneously by using the scheme presented in Fig. 8. The welding strategy for the silver nanowires was composed of two steps: (a) pre-treatment; multi-flash white light irradiation (energy: 50 J/cm^2^ to 70 J/cm^2^, pulse duration: 10 ms, irradiation time: 5 ms, pulse number: 15) with continuous UV-C light irradiation (2.78 mW/cm^2^) and (b) main welding; flash white light irradiation and main welding (energy: 26 J/cm^2^, irradiation time: 10 ms, pulse number: 1) with continuous UV-C light irradiation (2.78 mW/cm^2^).

The HPMC binder was efficiently evaporated in step (a) without embedding the silver nanowires into the PET film. After main flash white light irradiation with continuous UV-C irradiation (step (b)), the silver nanowires could be suitably welded and embedded into the PET substrate. To enhance the removal of organic binder for silver nanowire, the UV-C irradiation was adopted together with flash light irradiation. In general, the HPMC binder was thermally decomposed at the 220 °C and decomposed with UV-C irradiation for several hours^42^,^43^. Therefore, the HPMC binder around the silver nanowire could be decomposed due to high heat generated from the flash light with high intensity^42^. However, when the only flash white light was irradiated, the HPMC binder could not be fully decomposed owing to rapidly process time. These remained HPMC binders could be easily decomposed with both photo-thermal effect by flash light and photo-catalytic effect by UV-C light^42^,^43^. It was thus concluded that the use of UV-C light irradiation in the welding of the silver nanowires plays an important role in the evaporation of HPMC binder and embedding the silver nanowires into PET substrate. However, the main reason for the embedding of silver nanowire into PET substrate is the generated heat in the silver nanowire by flash light irradiation. In our experiments, the UV-C light was used for the effectively welding of silver nanowire as well as the decomposition of HPMC binder that help the silver nanowire embedded into the PET substrate. Therefore, UV-C light has the role of just assistant by decomposition of HPMC for the welding of the silver nanowire that evolved by flash light irradiation. Consequently, the sheet resistance of the silver nanowires welded by flash white light with continuous UV-C light irradiation was lower than that of the nanowires processed with flash white light irradiation only.

Successful welded and embedded silver nanowires films achieved by the optimal combined light welding process, shows an excellent mechanical reliability, transmittance (98.76%) and sheet resistance of a film (77.93 Ω/sq).

## Experiment Details

### Silver nanowire solution formulation

A silver nanowire (0.15 wt%, 20–30 nm in diameter, 30–40 μm in length, Nanotech and Beyond) solution was employed in this work. As an adhesive agent, HPMC binder (Sigma Aldrich) was dissolved in de-ionized (DI) water (99%; Samchun Chemical) by ultra-sonication for 1 h, and then added to the silver nanoparticle solution with stirring for 30 min; the weight fraction of HPMC binder was varied from 0 wt% to 0.3 wt%.

### Preparation of silver nanowire films

A polyethylene terephthalate (PET) film with a thickness of 225 μm was employed as a substrate in this study. To remove contaminants, the surface of the PET was cleaned in ethanol and distilled water using an ultra-sonicator for 10 min. The silver nanowire solution with HPMC binder was subsequently coated on the PET substrate by a bar-coating method. Drying of the silver nanowire films was carried out with an infrared lamp (NIR, wavelength range: 800~1500 nm, 500 W; Adphos L40) at a power of 350 W for 10 s.

### Combined light welding process

**Figure 1:**
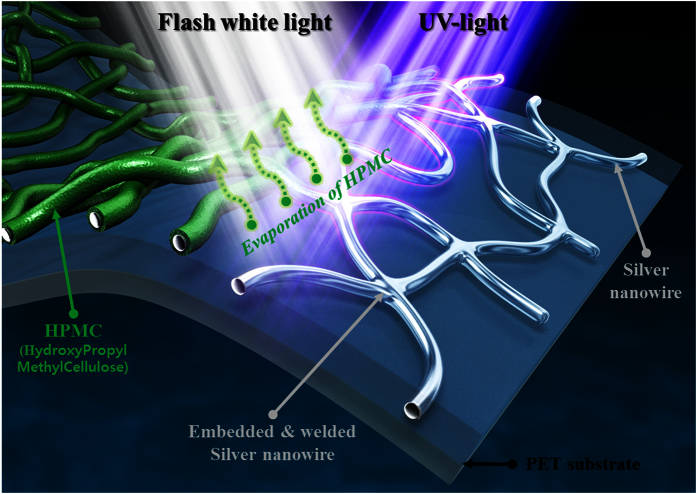
The schematic diagram for combined flash light welding process of silver nanowire with HPMC binder.

**Figure 2:**
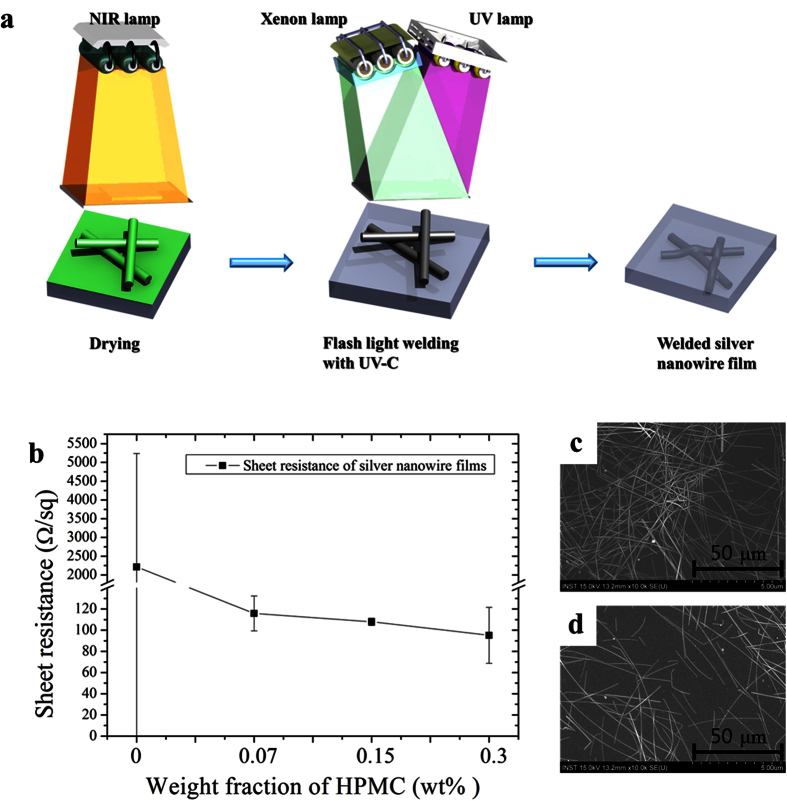
The coating uniformity for weight fraction of HPMC binder in the silver nanowire solution. (**a**) Schematic diagram of combined flash white light drying and welding process of silver nanowire, (**b**) The sheet resistance of silver nanowire films for weight fraction of HPMC binder and (**c,d**) the SEM image of silver nanowire films with 0.3 wt% in the other sheet resistance measurement point.

**Figure 3:**
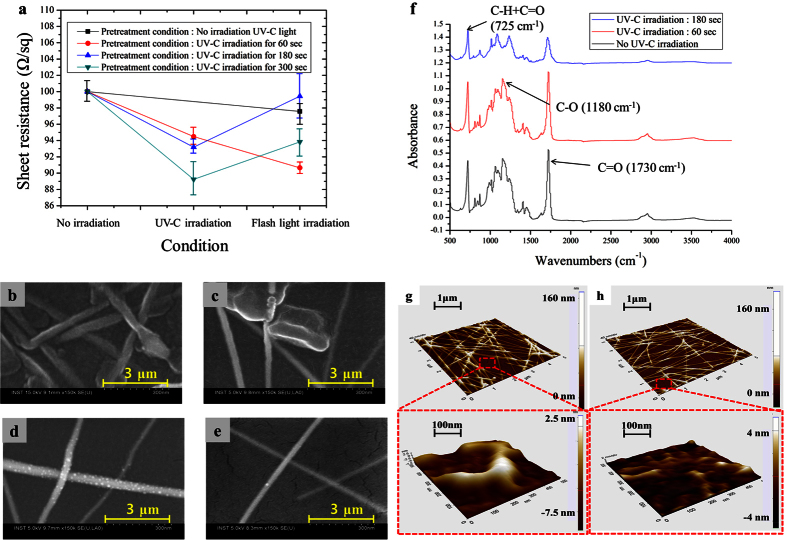
The effect of UV-C light irradiation time on the silver nanowires. (**a**) the sheet resistance of silver nanowire films with respect to UV light irradiation duration, the SEM image of silver nanowire films after UV-C light irradiation for (**b**) 0 sec, (**c**) 60 sec, (**d**) 180 sec, (**e**) 300 sec and (**f**) FT-IR spectra PET substrate for UV irradiation time and AFM image (**g**) before only UV irradiation and (**h**) after UV irradiation for 60 sec.

**Figure 4:**
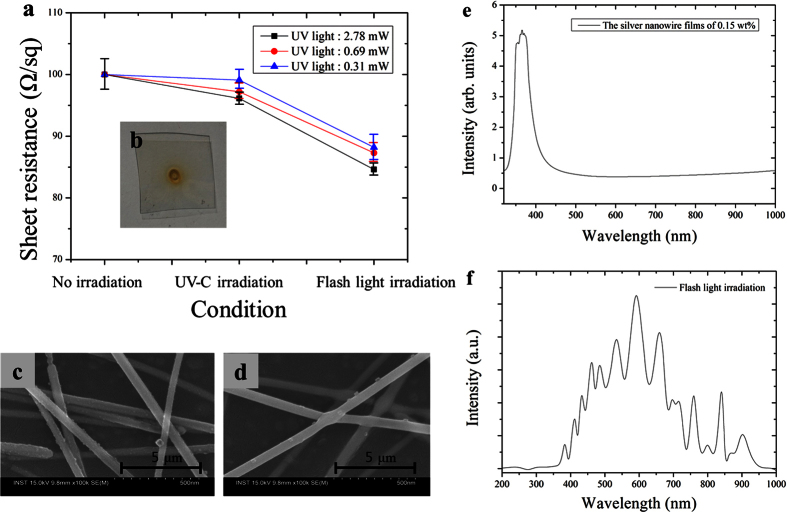
The effect of UV-C light irradiation energy on the flash light welding of silver nanowire. (**a**) the sheet resistance of silver nanowire films for UV irradiation energy, (**b**) the damage of silver nanowire films by the irradiated UV-C light with excessive energy (more than 2.78 mW/cm^2^) and the SEM image of (**c**) after UV-C light irradiation (energy : 2.78 mW/cm^2^) and (**d**) after flash white light irradiation, (**e**) the UV-vis spectra of the silver nanowire films and (**f**) spectrum wavelength of a flash white light from xenon lamp.

**Figure 5:**
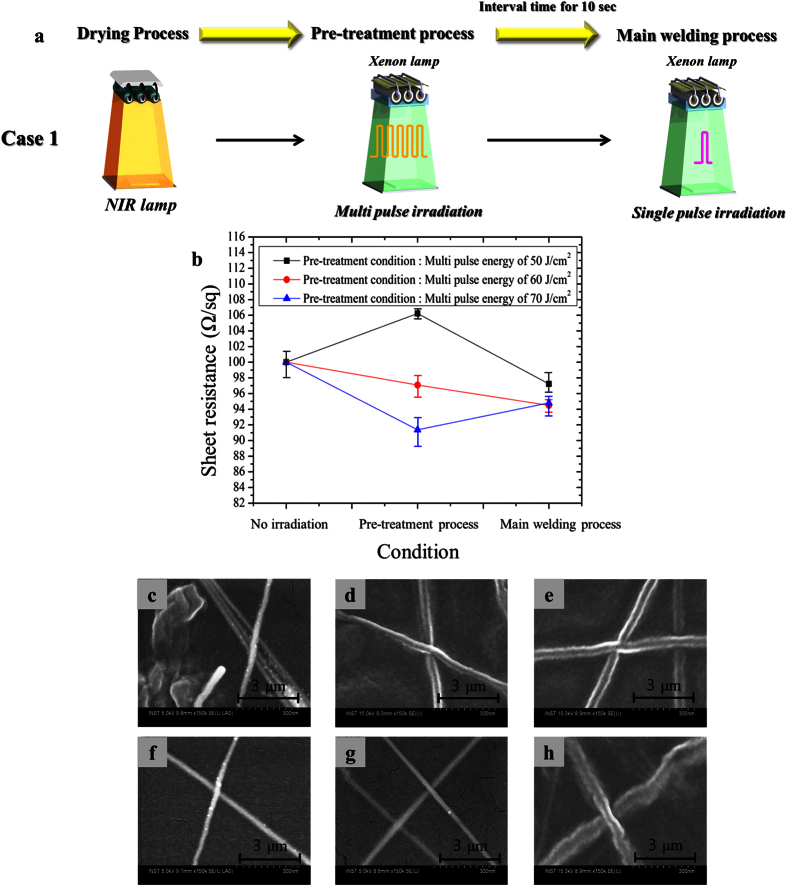
The effect of only the flash white light welding on silver nanowires. (**a**) The schematic diagram of experiment using only flash white light welding processes and (**b**) the sheet resistance of silver nanowires films with respect to multi pulse irradiation energy of the pre-treatment process (pulse number: 15, pulse duration: 10 ms, irradiation time: 5 ms) and the SEM image after pre-treatment process (**c–e**), after main welding process (**f**–**h**) ((**c**,**f**): pre-treatment energy 50 J/cm^2^, (**d**,**g**): pre-treatment energy 60 J/cm^2^, (**e**,**h**): pre-treatment energy 70 J/cm^2^).

**Figure 6:**
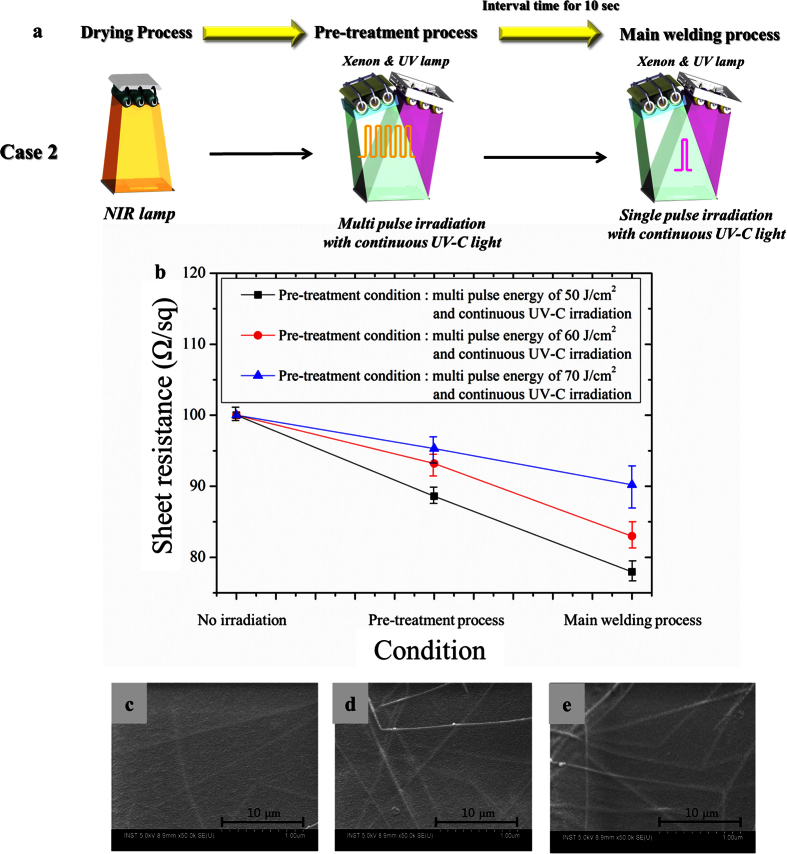
UV-C assisted flash light welding of silver nanowire. (**a**) The schematic diagram of experiment using combined flash white light welding processes and (**b**) the sheet resistance of silver nanowire films for irradiated multi purse energy with continuous UV-C light irradiation in the pre-treatment process (pulse number: 15, pulse duration: 10 ms, irradiation time: 5 ms) and the SEM image of welded silver nanowire films by main welding process with pre-treatment energy of (**c**) 50 J/cm^2^, (**d**) 60 J/cm^2^ and (**e**) 70 J/cm^2^.

**Figure 7:**
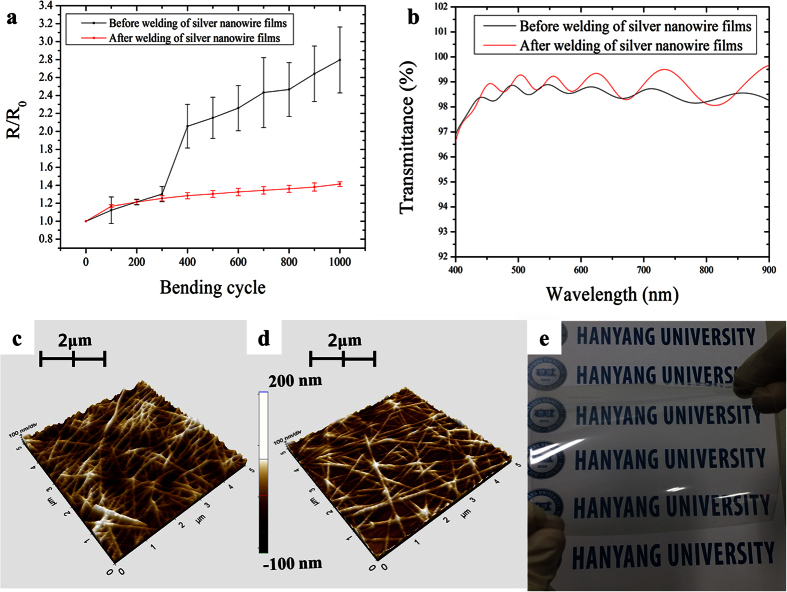
The characteristic evaluation of silver nanowire films before- and after- combined flash light irradiation. (**a**) The bending test to evaluated reliability before and after welding of silver nanowire films, (**b**) the transmittance before and after welding of silver nanowire films, the AFM data of surface morphology for silver nanowire (**c**) before combined flash light irradiation and (**d**) after optimized combined flash light irradiation and (**e**) the photograph of welded silver nanowire films by optimal combined welding condition.

**Figure 8:**
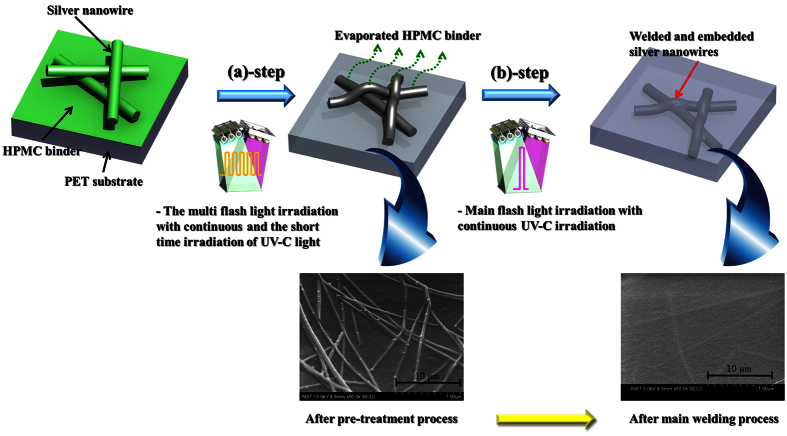
The schematic diagram for welding mechanism of silver nanowire using combined flash white light irradiation.

The coated silver nanowire films were welded at room temperature under ambient conditions using an in-house flash white light welding system and a UV-C light irradiation apparatus. The flash white light welding system consisted of a xenon lamp (Perkin-Elmer Co.), a power supply, capacitors, a pulse controller (Pstek Co.), and a reflector (Figs 1 and 2(a)). The light emitted from the xenon lamp had a wide range of wavelengths from 350 nm to 950 nm^25^,^26^,^27^,^28^. Here, the flash white light welding process was divided into pre-treatment and main welding steps. During the pre-treatment step, the flash white light irradiation energy was varied from 50 to 70 J/cm^2^, while the pulse duration, irradiation time, and pulse number were held constant at 10 ms, 5 ms, and 15, respectively. In the main welding step, the conditions of the flash white light welding process were fixed at an irradiation energy of 26 J/cm^2^, a pulse duration of 10 ms, and a pulse number of 1. To improve the welding characteristics for flash white light irradiation, a UV-C light irradiation system (UV-C, wavelength rage: 180~280 nm, 100 mW/cm^2^; LUMATEC SUV-DC) was utilized in both the pre-treatment and main welding steps. For the UV-C light irradiation procedure, the irradiation energy was varied from 0.31 to 2.78 mW/cm^2^, while the irradiation time ranged from 10 to 300 s.

### Characterization

The microstructure of the silver nanowire films was examined via scanning electron microscopy (SEM, S4800 HITACHI). Also, The sheet resistance of the silver nanowire films was measured with a four-point probe setup (tip spacing: 1 mm), while the transmittance of the films was evaluated with a spectrophotometer (Libra S70, Bruker Co.). To investigate the reliability of the silver nanowire films, the structures were bent repeatedly with a bending radius of 7 mm using a custom-built bending tester. Sheet resistances were also measured with respect to the bending cycle.

## Additional Information

**How to cite this article**: Chung, W.-H. *et al.* Welding of silver nanowire networks via flash white light and UV-C irradiation for highly conductive and reliable transparent electrodes. *Sci. Rep.* **6**, 32086; doi: 10.1038/srep32086 (2016).

## Figures and Tables

**Table 1:** the sheet resistance uniformity of silver nanowire films for weight fraction of HPMC binder.

The weight fraction of HPMC binder (wt%)	Measurement parts									Average	Standard deviation
	1	2	3	4	5	6	7	8	9		
**0**	2374	219.2	245.5	5202.0	341.9	269.6	2098.0	8937	243.5	2214.7	2851.765
0.07	146.5	110.5	102.8	122.6	94.25	99.99	123.7	129.9	112.1	115.86	15.56702
0.15	111.0	104.8	105.9	107.8	108.1	112.5	107.5	111.5	101.9	107.93	3.226411
0.3	89.04	101.8	97.49	110.1	78.26	155.7	74.03	78.35	71.32	95.145	24.87309
